# Supplementary material for: SynapseNet: Deep learning for automatic synapse reconstruction
Source: Mol Biol Cell. 2025 Sep 19;36(10):ar127. doi: 10.1091/mbc.E24-11-0519 (PMC12483319; doi:10.1091/mbc.E24-11-0519)
Supplement: Supplementary file 1 [file mbc-36-ar127-s001.pdf]

Supplemental Materials

Molecular Biology of the Cell

Muth *et al*.

SynapseNet: Deep Learning for Automatic Synapse Reconstruction: Supplementary Material

Supplementary Tables

EM Dataset		Sample Description			Sample Preparation / Image Acquisition		Data Usage		
**Name**	**Publication**	**Organism**	**Synapse**	**#**	**Fixation**	**Microscope**	**ST**	**DA**	**AS**
**Chemical Fixation**	*15*	Mouse	SC/MF	23	CF/HPF/AFS	200 kV JEM 2100 (JEOL)	SV/AZ M		
**Single-Axis TEM Tomo**	*12*	Mouse	SC	152	HPF/AFS	200 kV JEM 2100 (JEOL)	SV		
**Dual-Axis TEM Tomo**	*unpublished*	Mouse	HCC/MF	114	HPF/AFS	200 kV Talos F200C (Thermo)	SV		
**STEM Tomo**	*unpublished*	Mouse	SC/MF/PS	48	HPF/AFS	200 kV Talos F200C (Thermo)	SV/AZ M/SynC		
**IER**	*11,38,39 and unpublished*	Mouse Rat	IER	116	HPF/AFS	200 kV JEM 2100Plus (JEOL)	SR	SV	Fig. 6
**EH**	*unpublished*	Mouse	EH	170	HPF/AFS	200 kV JEM 2100Plus (JEOL)	AZ	SV	
**Cryo**	*unpublished*	Rat	HCC	22	PF	300 kV Krios G4 (Thermo)		SV	
**Frog**	*43*	Frog	NMJ	402	CF	80 kV CM10 (Philips)		SV	
**2D TEM**	*15*	Mouse	SC	13	HPF/AFS	80 kV LEO 912 (Zeiss)		SV	
**Munc13 / SNAP-25**	*12*	Mouse	SC	101	HPF/AFS	200 kV JEM 2100 (JEOL)			Fig. 4 Fig. 5

Supplementary Table 1: Summary of ultrastructural data used for supervised training and evaluation (yellow), domain adaptation (blue), and for application studies (red). Abbreviations: Data usage: Supervised training and evaluation, ST; domain adaptation, DA; Application study, AS. Synapse types and organelles: Schaffer collateral, SC; mossy fiber, MF; neuromuscular junction, NMJ; Endbulb of Held, EH; Inner ear ribbon synapses, IER; hippocampal cell culture, HCC; synaptic vesicles, SV; synaptic ribbons, SR; mitochondria, Mito; synaptic compartments, SynC. Fixation conditions: chemical fixation, CF; high-pressure freezing, HPF; automated freeze-substitution, AFS; plunge freezing, PF.

Supplementary Figures

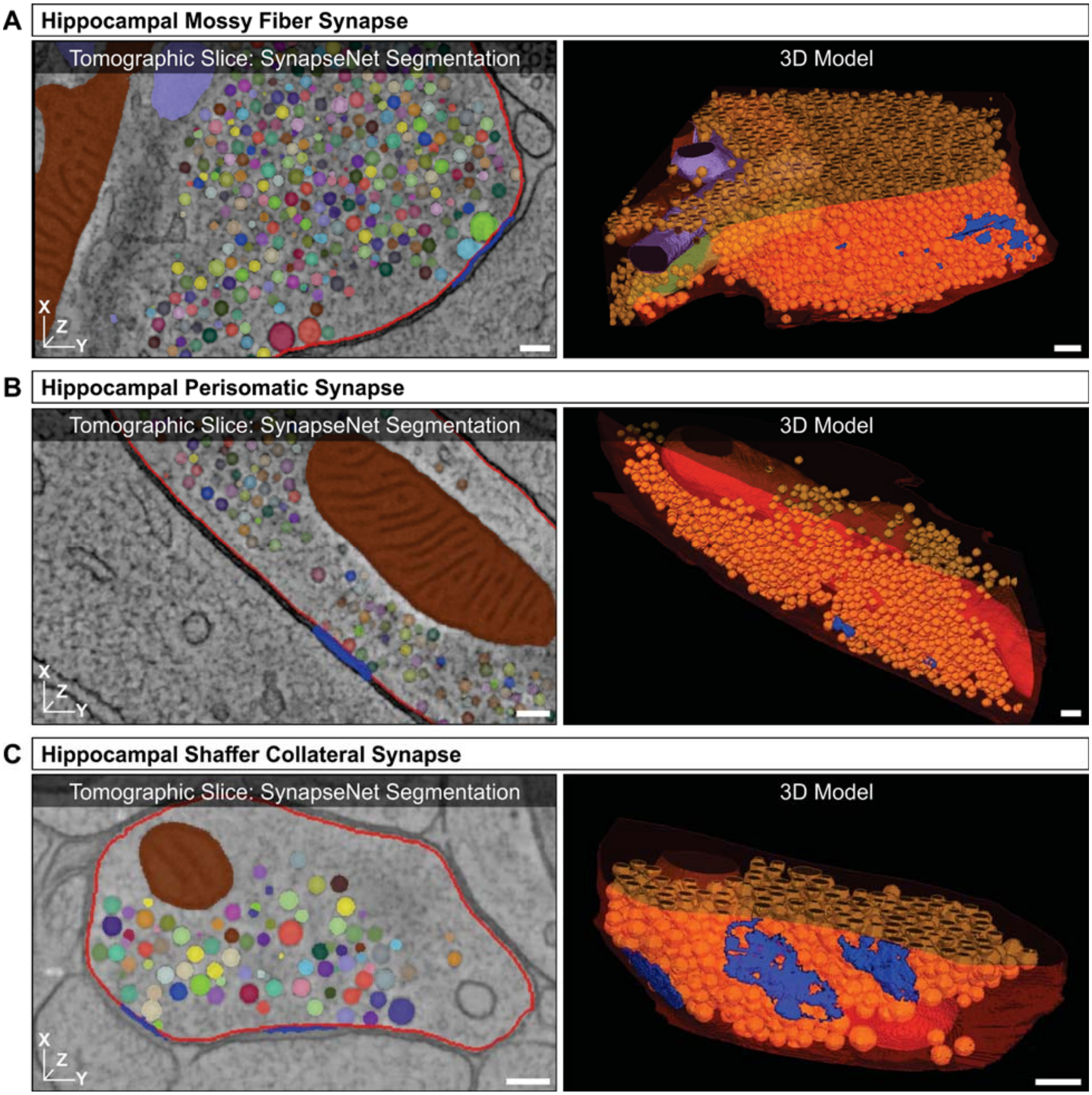

Supplementary Figure 1: Automated synapse reconstructions of a mossy fiber (A), perisomatic (B) and Schaffer collateral (C) synapse from SynapseNet. Active zones are rendered in blue, mitochondria in dark red and purple, synaptic compartments in red, and synaptic vesicles in a different color per object in the virtual section (left) and in orange in the 3D rendering (right). To obtain the reconstructions, different pieces (2-5) of the synaptic compartment predictions were merged for a correct segmentation, active zones were manually enlarged in some areas, all other segmentations are shown as they were predicted by the model. The scale bars represent 100 nm.

A 2D Synaptic Vesicle Segmentation		
**Dataset**	**SynapseNet**	
	F1 Score	SBD Score
**Chemical Fixation**	64.8%	49.5%
**Single-Axis TEM Tomo**	68.9 ± 2.2%	52.6 +- 3.1%
**Dual-Axis TEM Tomo**	77.4 ± 1.5%	63.5 +- 1.7%
**STEM Tomo**	69.5 ± 12.7%	60.3 +- 11.3%
**OVERALL**	70.7 ± 10.0%	58.8 +- 9.5%

B Active Zone Segmentation		
**Dataset**	**SynapseNet**	
	Surface Dice	Per Component
**Chemical Fixation**	74.3 ± 5.8	-
**Single-Axis TEM Tomo**	68.5 ± 20.0 %	-
**STEM Tomo**	48.9 ± 26.5%	71.9 ± 15.3%
**EH**	38.7 ± 17.7%	55.0 ± 26.0%

C Quantitative Evaluation of Mitochondria		
**Dataset**	**SynapseNet**	
	F1 Score	SBD Score
**STEM Tomo**	94.8 ± 5.1%	86.9 ± 7.1%

D Mitochondrial Segmentation

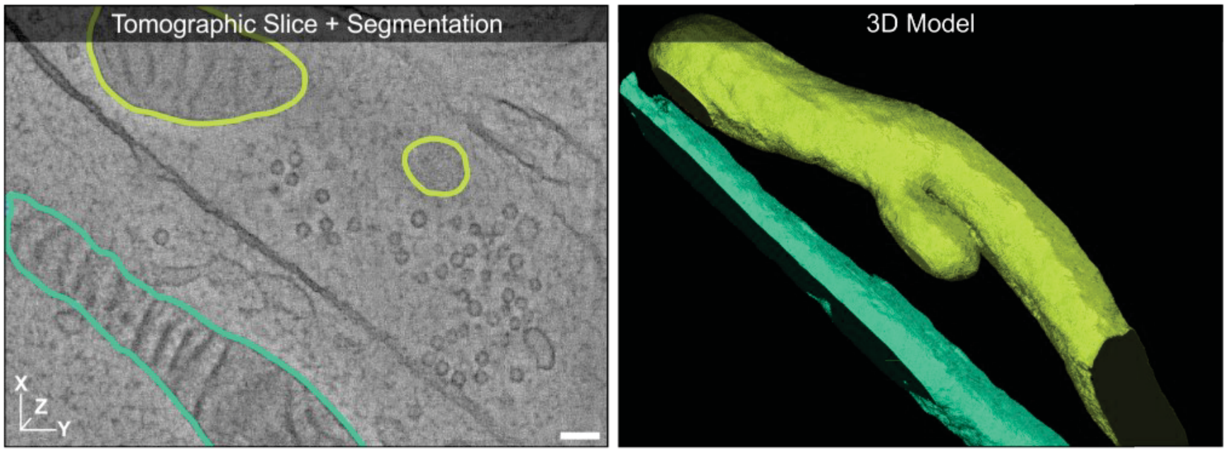

E Compartment and Active Zone Segmentation

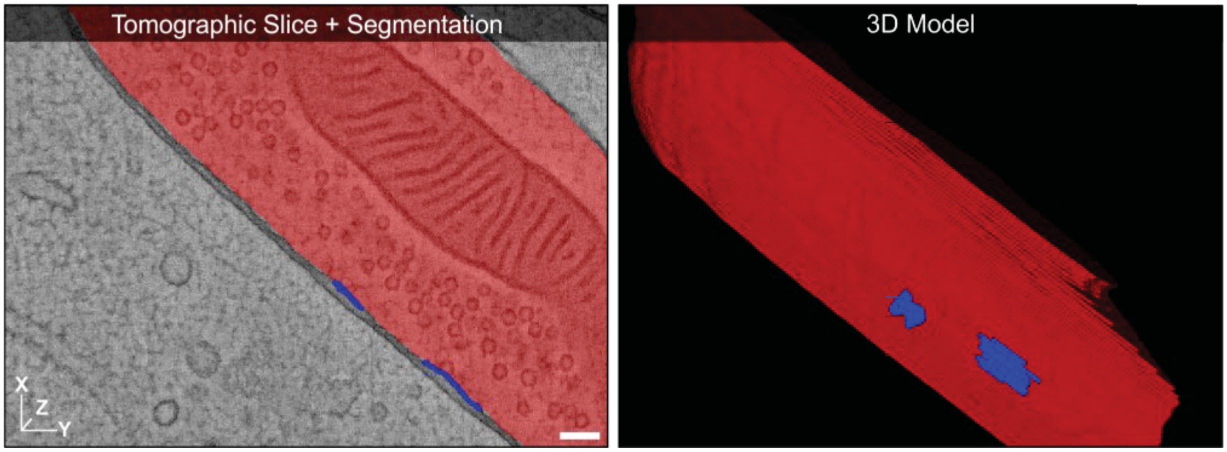

Supplementary Figure 2: Segmentation evaluation and examples. **A**. Evaluation of 2D synaptic vesicle segmentation with SynapseNet, using the same set-up as in Figure 2C, except for segmentation evaluation on individual sections instead of in 3D. Note that we don't provide a standard deviation for the chemical fixation setting due to the small number of tomograms. **B**. Evaluation of active zone segmentations. The surface Dice score is used for evaluation. The tomograms from STEM Tomo and EH are large and contain multiple active zones. For these cases, we also compute the surface Dice score for small crops around each active zone ("Per Component"), in order to evaluate the segmentation quality independent of false positives. **C**. Evaluation of mitochondrion segmentation. **D.** Example segmentations of mitochondria in a perisomatic synapse, for a virtual section (left) and rendered in 3D (right). Mitochondria are shown as overlays in red and cyan; and rendered in 3D in matching colors (right). **E.** Active zone (blue) and synaptic compartment (red) segmentations in a virtual section (left) and rendered in 3D (right) of a perisomatic synapse. All segmentations are unedited. The scale bars represent 100 nm.

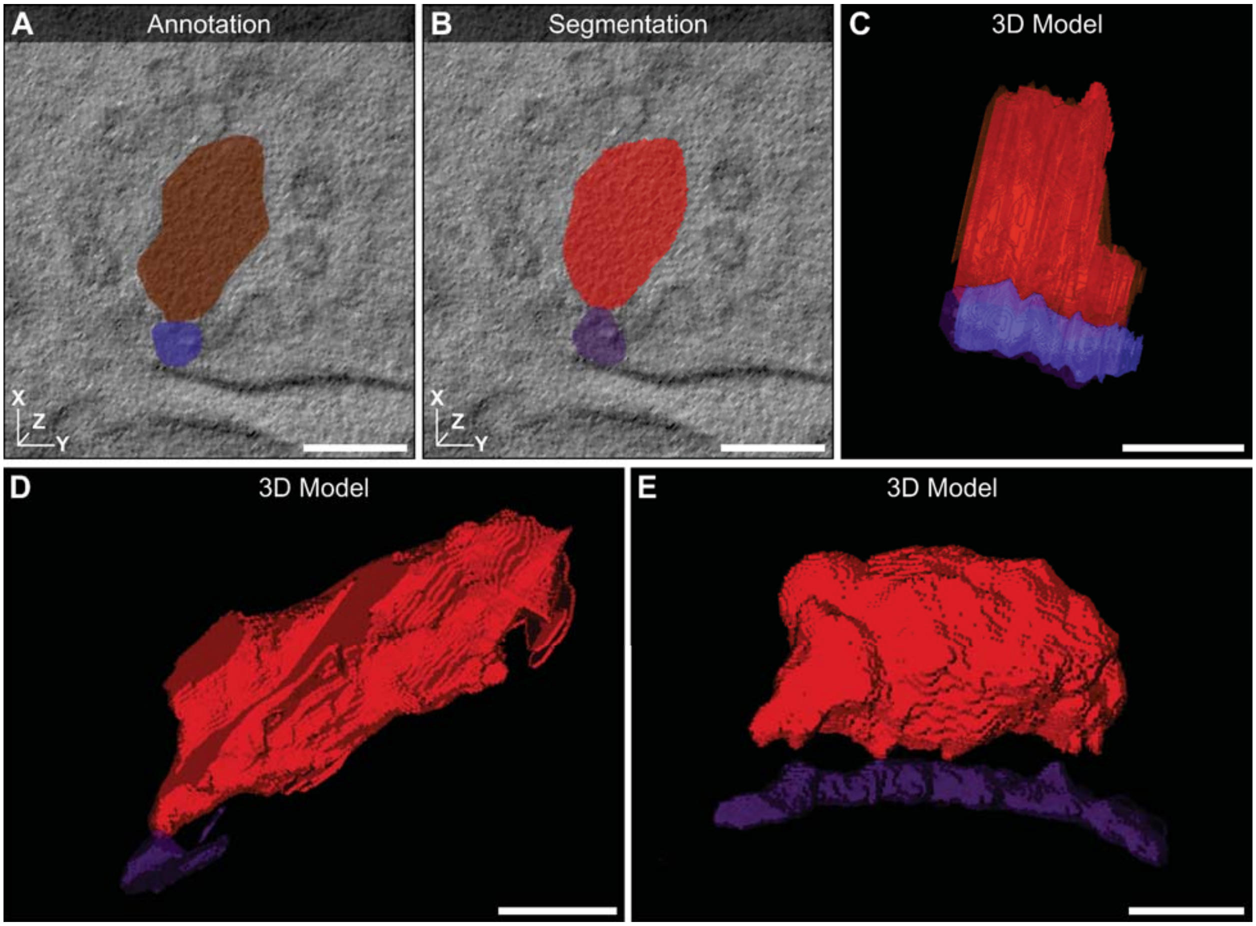

Supplementary Figure 3: Example segmentations for ribbons and presynaptic densities in inner ear ribbon synapses. **A**,**B**. Annotation and SynapseNet segmentation of synaptic ribbon (red) and presynaptic density (purple) overlaid on top of a virtual section. **C.** 3D rendering of annotation and segmentation on top of each other. **D**,**E.** 3D renderings of two other ribbon and presynaptic segmentations. The scale bars represent 100 nm.

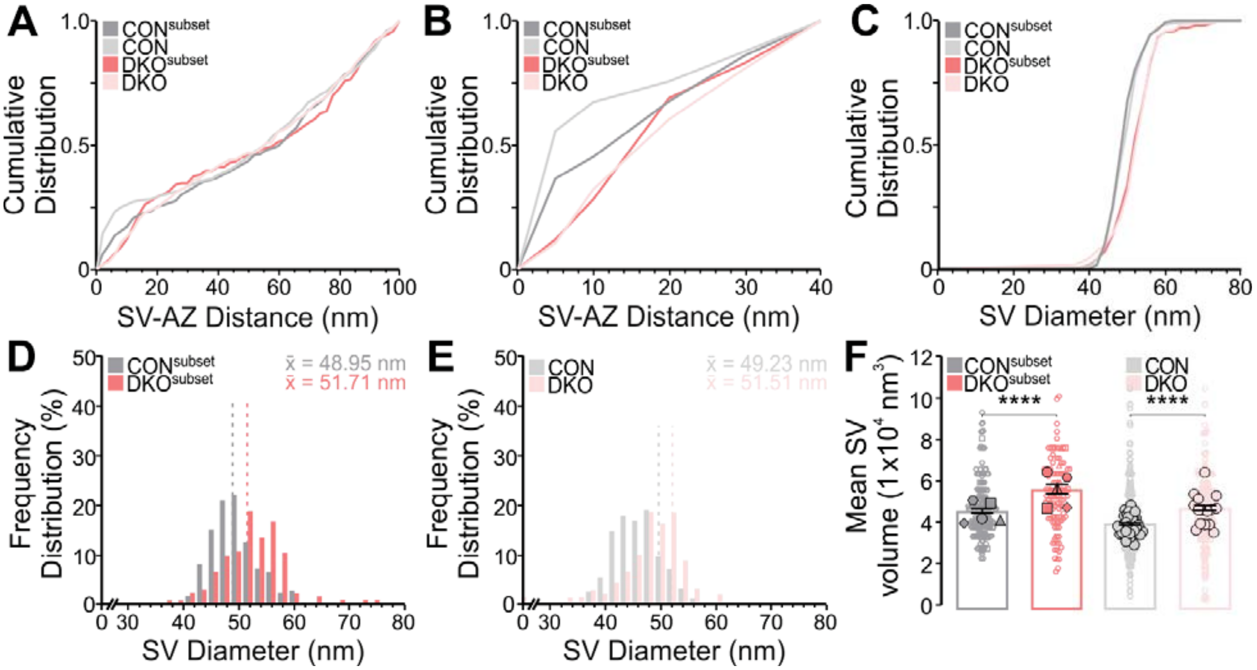

Supplementary Figure 4: 3D Electron tomographic analysis of synaptic vesicles in automatically segmented Munc13-1/2 double knock-out (DKO) and control (CON) hippocampal Schaffer collateral neurons. Analyses are based on automatic segmentation of synaptic vesicles (SVs) and semi-automatic segmentation of the active zone (AZ) from control and Munc13-1/2 DKO synapses. A subset of CON and 5 of DKO synapses (n=5) was compared with a larger dataset of electron tomographic subvolumes (CON, n=31; Munc13-1/2 DKO, n=15). **A**,**B**. Cumulative spatial distribution of SVs within 100 nm and 40 nm of the AZ. **C**. Cumulative distribution of SV diameters within 100 nm of the AZ. **D**,**E**. Frequency distribution of SV diameters within 100 nm of the AZ. Dotted lines indicate the mean SV diameter ($\bar{x}$). **F**. Scatterplot of the mean volume of SVs within 100 nm of the AZ. Filled data points indicate the mean SV volume of single ET subvolumes and empty data points with the same symbol shape indicate the volume of single SVs within the same ET subvolume. Values indicate mean ± SEM; ****$p < 0.0001$; ***$p < 0.001$; **$p < 0.01$; *$p < 0.05$.

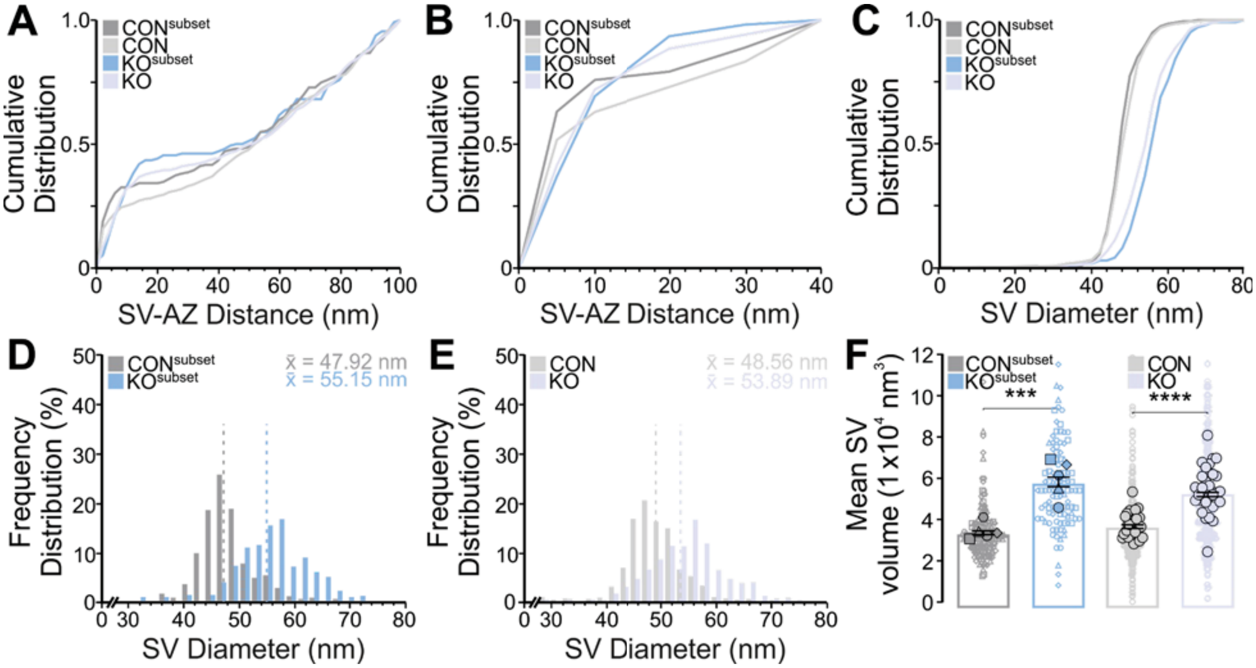

Supplementary Figure 5: 3D Electron tomographic analysis of synaptic vesicles in automatically segmented control (CON) and SNAP-25 knock-out (KO) hippocampal Schaffer collateral neurons. Analyses are based on automatic segmentation of synaptic vesicles (SVs) and semi-automatic segmentation of the active zone (AZ) from control and SNAP-25 KO synapses. A subset of CON (n=5) of KO synapses (n=5) was compared with a larger dataset of electron tomographic subvolumes (CON, n=27; SNAP KO, n=28). **A,B.** Cumulative spatial distribution of SVs within 100 nm and 40 nm of the AZ. **C.** Cumulative distribution of SV diameters within 100 nm of the AZ. **D,E.** Frequency distribution of SV diameters within 100 nm of the AZ. Dotted lines indicate the mean SV diameter ($\bar{x}$). **F.** Scatterplot of the mean volume of SVs within 100 nm of the AZ. Filled data points indicate the mean SV volume of single ET subvolumes and empty data points with the same symbol shape indicate the volume of single SVs within the same ET subvolume. Values indicate mean ± SEM; ****$p < 0.0001$; ***$p < 0.001$; **$p < 0.01$; *$p < 0.05$.

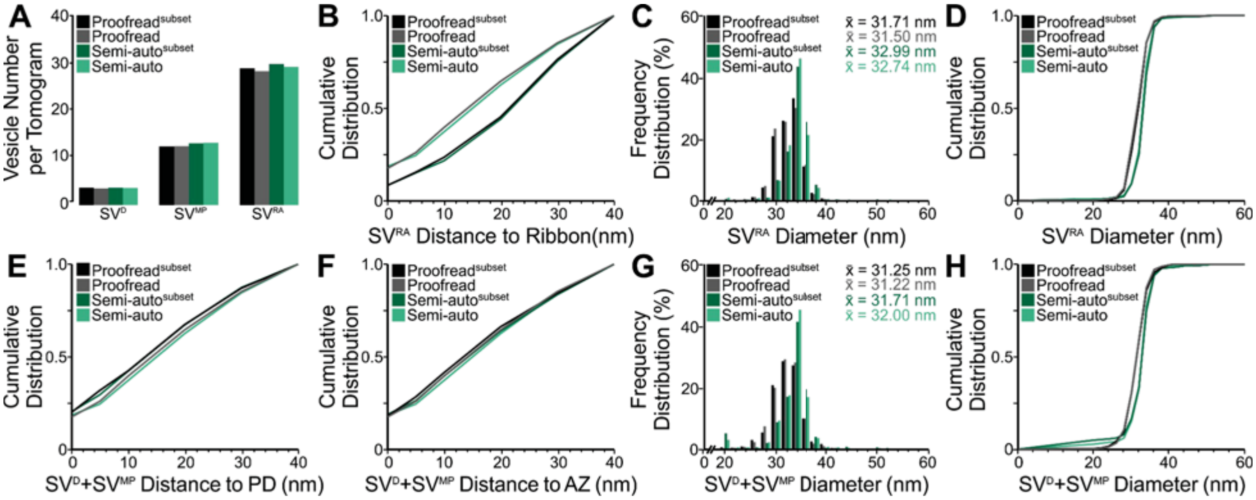

Supplementary Figure 6: Analysis of ribbon synapses of cochlear inner hair cells in the inner ear. A subset (n=33) and a larger dataset of proofread and semi-automatic segmentations (n=88) were analysed. **A.** Average number of docked synaptic vesicles (SV^{D}), membrane-proximal SVs (SV^{MP}), and ribbon-associated SVs (SV^{RA}) per tomogram. **B.** Cumulative spatial distribution of SV^{RA} within 40 nm of the ribbon. **C,D.** Frequency distribution of SV^{RA} diameters with the mean SV diameter ($\bar{x}$) and cumulative distribution of SV diameters. **E,F.** Cumulative spatial distribution of SV^{D}+SV^{MP} within 40 nm of the PD and the AZ. **G,H.** Frequency distribution of SV^{D}+SV^{MP} diameters with the mean SV diameter ($\bar{x}$) and cumulative distribution of SV diameters. Values indicate the mean diameter.

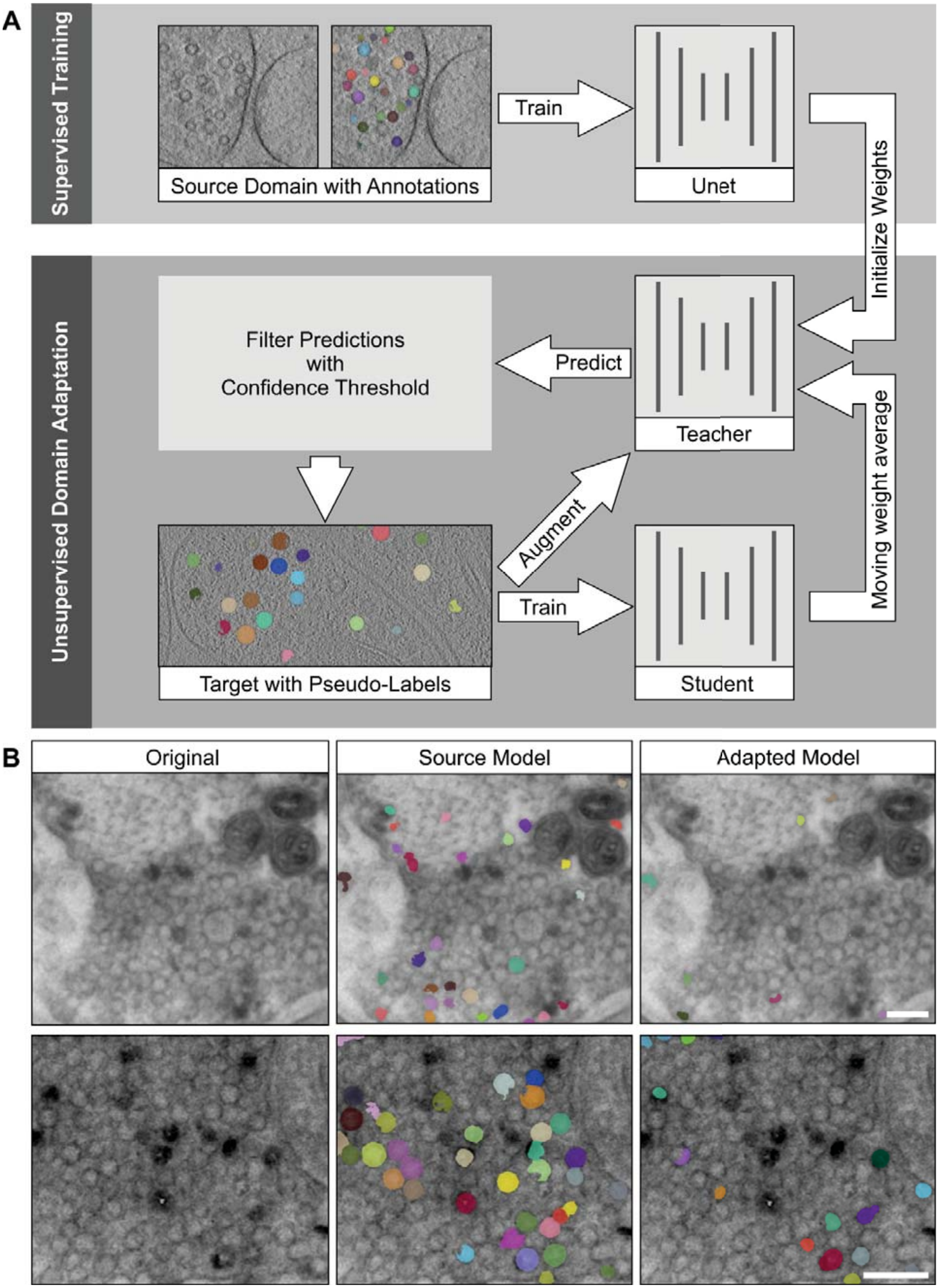
A
Supervised Training
Source Domain with Annotations
Train
Unet
Initialize Weights
Unsupervised Domain Adaptation
Filter Predictions
with
Confidence Threshold
Predict
Teacher
Moving weight average
Augment
Target with Pseudo-Labels
Train
Student
B
Original
Source Model
Adapted Model

Supplementary Figure 7: **A.** Schematic overview of domain adaptation. To transfer a model to a new domain (different imaging modality, sample preparation, specimen, etc.), we start from a model trained on the source domain, corresponding to the model trained via supervised training on data with annotations (top). For training on the target domain the model architecture is duplicated to obtain a teacher and a student model. In each training iteration the teacher model is applied to an augmented version of the image and its output is filtered to retain only confident predictions. The student model is applied to the image and its predictions are compared via a loss function to the filtered teacher output. The weights of the student network are updated via stochastic gradient descent (or a variant thereof) and the weights of the teacher network are updated via exponential moving average of the student network's weights. **B.** Limitations of domain adaptation. If the initial predictions of the teacher network miss the majority of objects, e.g. vesicles, then the domain adaptation will likely fail and converge to predicting fewer or even no objects at all. This is the case for the frog data, which is shown here. The appearance of the image data is too different from the annotated vesicle training data due to lower contrast, leading to bad predictions of the source model and worse predictions after domain adaptation. The scale bars represent 200 nm.

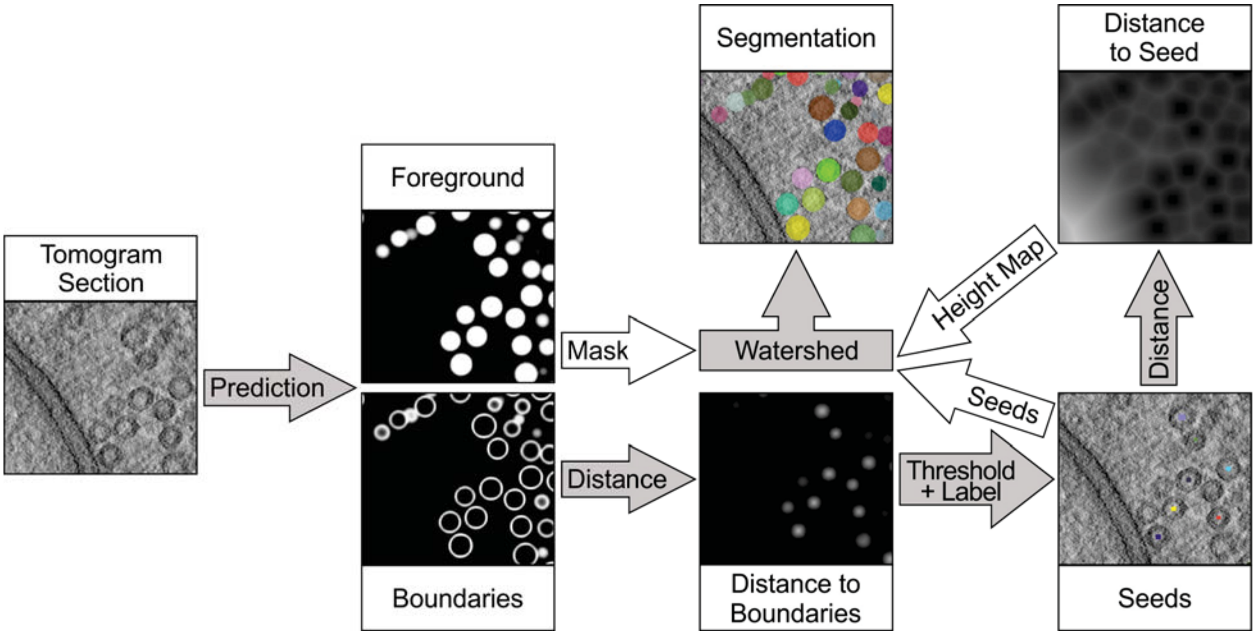

Supplementary Figure 8: Watershed-based instance segmentation logic for synaptic vesicles. The network predicts probability maps for foreground and boundaries ("Prediction") based on the tomogram data. The watershed is run based on seeds, a height map, and a mask. The seeds are derived from the boundary prediction by first computing a distance transform of the thresholded boundary predictions, and then applying a threshold to this distance map, which is labeled via connected components. The height map is derived from a distance transform computed based on the (binarized) seeds. The mask is derived from the foreground prediction by applying a threshold.
