# Supplementary material for: SynapseNet: Deep learning for automatic synapse reconstruction
Source: Mol Biol Cell. 2025 Sep 19;36(10):ar127. doi: 10.1091/mbc.E24-11-0519 (PMC12483319; doi:10.1091/mbc.E24-11-0519)
Supplement: Supplementary file 2 [file reviewer_reportE24-11-0519.pdf]

SynapseNet: Deep Learning for Automatic Synapse Reconstruction

Sarah Muth, Frederieke Moschref, Luca Freckmann, Sophia Mutschall, Ines Hojas-Garcia-Plaza, Julius Bahr, Arsen Petrovic, Thanh Thao Do, Valentin Schwarze, Anwai Archit, Kirsten Weyand, Susann Michanski, Lydia Maus, Cordelia Imig, Anika Hintze, Nils Brose, Carolin Wichmann, Ruben Fernandez-Busnadiego, Tobias Moser, Silvio Rizzoli, Benjamin Cooper, and Constantin Pape

Corresponding author(s): Constantin Pape, Georg-August-Universität Göttingen

Review Timeline:

Submission Date:	2024-12-02
Editorial Decision:	2025-02-04
Revision Received:	2025-07-23
Accepted:	2025-07-24

Editor-in-Chief: Matthew Welch

Transaction Report:

(Note: With the exception of the correction of typographical or spelling errors that could be a source of ambiguity, letters and reports are not edited. The original formatting of letters and referee reports may not be reflected in this compilation.)

RE: Manuscript #E24-11-0519
TITLE: SynapseNet: Deep Learning for Automatic Synapse Reconstruction

Dear Constantin,

Please let me begin by apologizing for our delay reviewing your paper. We were waiting on one reviewer who was late with their review submission.

You will see that both reviewers are positive. Reviewer #1 have several specific comments that you should consider regarding the evaluation metrics and potential leakage that, as far as I can tell, can be addressed with more clarification and some analyses. Reviewer #2 raised several points that are worth elaborating on in the Discussion. In addition, I recommend providing more information in the Results, specifically moving Supp. Fig. 7A and providing more intuition regarding the domain adaptation in the Results. Please also write a Significance Statement according to the instructions https://www.molbiolcell.org/curation-tools. Do not hesitate to contact me if there are specific requests from the reviewers that you believe are not necessary or unlikely to yield a meaningful outcome.

I look forward to receiving your revised manuscript, together with a letter indicating the changes you've made and your responses to the referees.

Best,
Assaf

Assaf Zaritsky
Monitoring Editor
Molecular Biology of the Cell

Dear Dr. Pape,

The review of your manuscript, referenced above, is now complete. The Monitoring Editor has decided that your manuscript is not acceptable for publication at this time, but may be deemed acceptable after specific revisions are made, as described in the Monitoring Editor's decision letter above and the reviewer comments below.

A reminder: Please do not contact the Monitoring Editor directly regarding your manuscript. If you have any questions regarding the review process or the decision, please contact the MBoC Editorial Office (mboc@ascb.org).

When submitting your revision include a rebuttal letter that details, point-by-point, how the Monitoring Editor's and reviewers' comments have been addressed. (The file type for this letter must be "rebuttal letter"; do not include your response to the Monitoring Editor and reviewers in a "cover letter.") Please bear in mind that your rebuttal letter will be published with your paper if it is accepted, unless you have opted out of publishing the review history.

Authors are allowed 90 days to submit a revision. If this time period is inadequate, please contact us at mboc@ascb.org.

Revised manuscripts are assigned to the original Monitoring Editor whenever possible. However, special circumstances may preclude this. Also, revised manuscripts are often sent out for re-review, usually to the original reviewers when possible. The Monitoring Editor may solicit additional reviews if it is deemed necessary to render a completely informed decision.

In preparing your revised manuscript, please follow the instruction in the Information for Authors (www.molbiolcell.org/info-for-authors). In particular, to prepare for the possible acceptance of your revised manuscript, submit final, publication-quality figures with your revision as described.

To submit the rebuttal letter, revised manuscript, and figures, use this link: Link Not Available

MBoC offers the option to publish your work with immediate open access. Open access can increase the discoverability and usability of your research. If you would like to publish your paper with immediate open access but did not select this option during initial submission, please contact the editorial office at mbc@ascb.org.

Authors of Articles and Brief Communications are also encouraged to create a short video abstract to accompany their article when it is published. These video abstracts, known as Science Sketches, are up to 2 minutes long and will be published on YouTube and then embedded in the article abstract. Science Sketch Editors on the MBoC Editorial Board will guide you as you

prepare your video. Information about how to prepare and submit a video abstract is available at www.molbiolcell.org/science-sketches. Please contact mboc@ascb.org if you are interested in creating a Science Sketch.

Sign up for MBoC's new content alerts here https://tinyurl.com/3bcsyk3e

Thank you for submitting your manuscript to MBoC. We look forward to receiving your revised paper.

Sincerely,

Eric Baker
Journal Production Manager
MBoC Editorial Office
mbc@ascb.org

Reviewer #1 (Remarks to the Author):

Muth et al. present SynapseNet, a tool that uses deep learning to automate the segmentation and analysis of synaptic structures (synaptic vesicles, active zones, mitochondria, and presynaptic compartments) in electron tomograms of resin-embedded samples. To date, such analyses have mostly been performed with the use of manual segmentation, which is a significant bottleneck. This is especially the case for the segmentation synaptic vesicles, which are present in hundreds to thousands of copies in a synapse. The authors have assembled a large annotated dataset for training. They use U-Net models. The strategy to perform instance segmentation of densely packed synaptic vesicles is elegant. It combines a foreground model and a boundary model, as well as postprocessing steps, to maxmize the accuracy of individual vesicle identification, even in crowded regions. Muth et al. introduce an unsupervised domain adaptation strategy based on a teacher model and a student model. This technique is effective at improving segmentation performance across various types of synapses and types of electron microscopy techniques, such as Cryo-ET. Finally the authors present a napari plugin that makes it possible to inspect and correct annotations. SynapseNet is an exciting tool with the potential to accelerate progress in synaptic research.

Nonetheless, I have some concerns, which should be addressed.

My main concerns primarily relate to the evaluation of segmentation performance and its quantitative comparison with other methods.

Synaptic vesicle segmentation performance is evaluated with the F1-Score measured at the intersection over union of 50%. While a high F1-Score indicates that issues such as merging or splitting of vesicle are minimal, the relatively low threshold may fail to capture finer errors, particularly along the edges of the labels, where segmentation accuracy is most critical for precise morphological analysis. In addition to F1-Score, the authors should use the Dice coefficient. They should also use a metrics to evaluate how accurately the surface of the predicted labels matches the surface of the ground-truth labels, such as surface Dice coefficient.

On page 20, it is stated that the final training data for vesicle segmentation contained 117K annotations, of which 80K derived from manual annotations, and 37K are based on automatically segmented vesicles. More than 30% of the labels used both for training and for testing were generated by SynapseNet. This could bias the process and should ideally be avoided. The authors should evaluate the performance of synaptic vesicle segmentation trained solely on the 80K manual annotations. Additionall, they should assess the performance of the model trained with the 117K annotations, but the evaluation should be restricted to the subset of vesicles that were manually annotated.

The authors compare synaptic vesicle segmentation performance of SynapseNet and CryoVesNet. It is not clear how they used CryoVesNet. Did they use only the U-Net or did they also use the postprocessing steps of CryoVesNet? This should be clarified. CryoVesNet has been trained on a cryo-ET dataset. It is therefore not surprising that it performs much worse on resin-embedded samples than SynapseNet, as the authors write it. It should also be mentioned that the size of CryoVesNet training dataset (1369 vesicles) is about 100 fold smaller than SynapseNet training dataset and that CryoVesNet was trained with fewer epochs. In order to ensure a fair comparison, I propose two options: either omit the F1-Scores of CryoVesNet for resin-embedded samples from Figure 2B and Figure 3A for EH and IER, or retrain CryoVesNet using the same training dataset as SynapseNet and include a third column in Figure 2B to present the F1-Scores of the retrained model. Another interesting comparison could be to train SynapseNet with the training set of CryoVesNet (available on EMPIAR, including the training labels) and evaluate its performance on cryo-ET datasets.

It would be interesting to compare the performance of synaptic vesicle segmentation of SynapseNet with 3DART-VeSElecT, which is based on classical image analysis. The authors decided not to do it because it requires some manual parameter tuning, which prevents automation. Nonetheless, the authors could test 3DART-VeSElecT with its default parameter values.

Furthermore Gomez-de-Mariscal et al. have developed a deep-learning-based segmentation of small extracellular vesicles in TEM images (https://doi.org/10.1038/s41598-019-49431-3). It would be also interesting to compare the performance of this approach with SynapseNet for synaptic vesicle segmentation.

The segmentation of mitochondria and synaptic compartments were only visually evaluated. This is not sufficient. F1-score and Dice coefficient should be presented.

The benefit of active zone segmentation si not very clear. Dice coefficient is relatively poor (~0.5). On another test set, it is stated that 67% of active zones were found, but in this case, there is no mention of the false positive rate. Could the authors specify an F1-Score? On page 10, it is stated that in 60% of active zones were segmented completely and 40% had to be corrected. Figure 5 compares manually segmented active zones (Fig. 5B and E) with semi-automatically segmented active zones (Fig. 5C and F). They look visually identical but it would be interesting to see the fully automatically segmented AZ for comparison. The authors could indicate how much time/effort is saved by the semi-automatic segmentation of AZ versus fully manual segmentation.

The discussion states : "This contribution marks the first comprehensive approach for automatic synapse reconstruction from electron micrographs". Although one aspect of the synapse reconstruction is fully automatic (vesicle segmentation), most other aspects are not. Indeed, in addition to active zone segmentation, other structures are semi-automatically segmented: synaptic compartments (mostly split in multiple pieces), presynaptic density in ribbon synapses and to some extent synaptic ribbons. Therefore this sentence, as well as several other sentences, including the title should be modified to reflect the reality of the method.

The flow of the manuscript can be difficult sometimes. Many abbreviations used throughout the manuscript (such as IER, EH, ...) are introduced in the Materials and Methods section, which comes at the end of the manuscript, making it difficult to follow the text seamlessly. If the journal permits, it would be helpful to place the materials and methods section before the results section.

Minor comments

p.5 "A detailed description of all underlying data is given in Sample Preparation and Data Acquisition...". If the materials and methods section does not preced the results section, it would be helpful to briefly mention the underlying data here to improve the reading flow.

In the results section, the description of the different modalities, e.g. in figure 2 B, C, and D, chemical fixation vs single-axis tomo, is confusing. Indeed as far as I understand, "chemical fixation" refers to chemically fixed samples, which were then imaged by single-axis tomography.

In the legend of figure 2, it would be helpful to mention which samples are shown (which types of synapse).

In the legedn of figure 3, it would be helpful to mention which evaluation metrics is shown.

P. 10, Figure 4I: p=0.016; Figure fI: p=0.032). Figure fI is a mistake.

P. 10. "Such discrepancies likely reflect the fact that only a smaller randomly selected subpopulation of synapses were sampled from the full datasets previously analyzed (Imig et al., 2014)." If I understand correctly it means that a smaller dataset has been analysed after automatic segmentation than the originally manually segmented data? Why not analyse the exact same dataset as in the original study but with automatic segmentation?

Figure 6. Some of the X-axis labels are too small.

P. 17. "Most of the data is room temperature electron tomography, but we also included cryogenic electron tomography and serial section transmission electron microscopy." It is not very clear that cryo-ET and ssTEM data are used only for domain adaptation.

Pixel sizes should be indicated in the data acquisition descripitions.

P. 22. VesiclePicker is not used to automatically segment synapses. It is used to segment vesicles in cryo-EM micrographs (2D images).

P. 23. "The networks were for 100,000 iterations and the checkpoint of the epoch with the lowest validation loss was exported." "run" is missing after the networks were. 100,000 epochs is very large. At which iteration was typically the lowest validation loss? If it was early, what was the rational for running so many epochs?

P. 23. It would be helpful to illustrate graphically the steps of synaptic vesicle segmentation. Showing an real life example of

tomogram region with synaptic vesicles, next to the corresponding prediction maps (boundary and foreground), distance maps, heightmaps, and final results.

P. 26. How many iterations are performed in the domain adaptation? How computationally intensive is the domain adaptation?

Ref 30 has been published.

Suppl. Table 1. Define SynC in the legend.

Suppl. Figure 2A. No standard deviation is given for chemical fixation. In the legend of this figure red and blue should be swapped (active zone / synaptic compartment)

Suppl Figure 5E and 6E are faint (transparent)

Reviewer #2 (Remarks to the Author):

This manuscript introduces SynapseNet, a tool for the segmentation of synaptic compartments and subcellular structures in electron microscopy volumes, which is based on a pre-trained neural network. The tool is available as a napari plugin, which also provides some basic analysis features. Furthermore, SynapseNet provides a method to transfer the pre-trained network in an unsupervised fashion to EM datasets that are not representative of the training dataset. The datasets used to train SynapseNet are publicly available for dataset sources that have already been published (and others will be made available once their corresponding publications are).

The pre-trained network itself is very relevant for the analysis of large EM volumes of neural tissue, which are now routinely acquired. The napari plugin and python package further lower the bar to using this method, especially with the ability to adapt to a new domain (albeit with mixed results, which is somewhat expected). In the long run, the provided datasets will be very useful to train future networks. The manuscript itself is very well written, provides sufficient technical details to reproduce the findings, and the claims made are sufficiently backed up by a series of exhaustive experiments and evaluations.

I recommend acceptance of this manuscript and have only minor comments.

* From reading the manuscript, it was not entirely clear whether the Domain Adaptation component is also part of the napari plugin. After reading the documentation, it seems it is not. I suggest that the authors make the distinction between SynapseNet (the method and pre-trained network) and the tool (the napari plugin) more clear. A similar point can be made regarding the fine-tuning (although I do acknowledge that the manuscript mentions that this feature is only available programmatically). Along those lines, I'd be curious to hear whether there are plans to include both features in the plugin, or whether there are practical concerns that prevent it.

* Regarding the Domain Adaptation, I wonder if the available ground-truth annotations could be leveraged to guide the student network, e.g., through an adversarial loss that criticizes solutions that do not look representative of real synapse annotations. Was a strategy like this considered, and if so, are there some preliminary findings that would be worth discussing?

* Are there any plans to consolidate a large synapse annotation dataset sourced from several contributors in the community? It seems like the authors are in an excellent position to start such an initiative, given the curated datasets they already have.

Dear Editor, dear Reviewers,

We have now revised our manuscript based on the reviews we received.

Before going into details about the revision, we would like to point out that after publication of our preprint (at the same time as the submission of the manuscript), we have seen significant interest in the use of SynapseNet for the analysis of synapses in electron microscopy. Notably, it was already used to segment synaptic vesicles in a large dataset of synaptosomes that were imaged in cryogenic electron tomography and that are available on the CZII Cryo ET Data Portal. The corresponding segmentation was produced by our domain adaptation method trained on a subset of synaptosome tomograms. The resulting vesicle segmentations are available at https://cryoetdataportal.czscience.com/depositions/10330. Furthermore, we have already used SynapseNet for data analysis in two studies from our research consortium, for analyzing synaptic vesicles in cryogenic electron tomography and for analyzing them in room temperature electron tomography of otoferlin knock-out mice for a study on the structure and function of otoferlin.

For the revision we have aggregated new annotated data for active zones and mitochondria, which we then used to improve and thoroughly evaluate the active zone model, and to thoroughly evaluate the mitochondria model. We have added a new co-author, Anika Hintze, to the manuscript, who has contributed some of the active zone annotations. We have addressed several other points raised by the reviewers, see the detailed responses to the editor and the reviewers below. Please note that this revision required a substantial amount of work due to the effort required to aggregate new data, provide additional annotations, and to train and evaluate the respective models.

We strongly believe that our manuscript now meets the criteria for publication and that the use of our method in other studies has already shown its value. We hope that you concur with this assessment.

We have marked all significant changes in the revised version of the manuscript in red.

Response to the editor:

> *Reviewer #1 have several specific comments that you should consider regarding the evaluation metrics and potential leakage that, as far as I can tell, can be addressed with more clarification and some analyses.*

We have addressed the majority of points raised by Reviewer #1. In particular, we clarified questions about the evaluation metrics and performed additional analyses to demonstrate that our procedure for generating training data for synaptic vesicles, which involves curated results from automated segmentation, is required to obtain good segmentation results. Furthermore, we have added more annotated data for the active zone and mitochondria segmentation to address the points raised by Reviewer #1 regarding these models. See the detailed response to

Reviewer #1 for more information and clarification as well as additional experiments regarding several other requests.

> *Reviewer #2 raised several points that are worth elaborating on in the Discussion. In addition, I recommend providing more information in the Results, specifically moving Supp. Fig. 7A and providing more intuition regarding the domain adaptation in the Results.*

We have addressed the points about advanced domain adaptation techniques and data aggregation raised by Reviewer #2.

> *Please also write a Significance Statement according to the instructions https://www.molbiolcell.org/curation-tools. Do not hesitate to contact me if there are specific requests from the reviewers that you believe are not necessary or unlikely to yield a meaningful outcome.*

We have written a significant statement. It can be found after the abstract.

Response to Reviewer #1:

> *Synaptic vesicle segmentation performance is evaluated with the F1-Score measured at the intersection over union of 50%. While a high F1-Score indicates that issues such as merging or splitting of vesicle are minimal, the relatively low threshold may fail to capture finer errors, particularly along the edges of the labels, where segmentation accuracy is most critical for precise morphological analysis. In addition to F1-Score, the authors should use the Dice coefficient. They should also use a metrics to evaluate how accurately the surface of the predicted labels matches the surface of the ground-truth labels, such as surface Dice coefficient.*

We have chosen the F1-score at an intersection over union of 50% due to the nature of annotations exported from IMOD, which are used for our training data: the vesicles were annotated as spheres, which are only an approximation of the true shapes. In contrast, the model predictions are not spheres, but segmentation masks, so they can better match to the actual shapes. Thus matching at a higher IOU may penalize correct predictions. Further, note that we deal with an *instance segmentation* problem. The Dice coefficient is a metric for *semantic segmentation*, and thus not directly suitable for this evaluation. In the revised version of the manuscript we have now included the “symmetric best dice score” (SBD, introduced in https://ieeexplore.ieee.org/document/9423433) as an additional metric for the evaluation of vesicle segmentation quality (Figure 2C, Figure 3A, Supp. Fig S2A). The SBD extends the dice coefficient to instance segmentation and does not suffer from fixed threshold effects, so it is a valid additional metric for this problem. Note however, that the SBD may underestimate the “actual” segmentation quality due to the spherical nature of annotations mentioned before. Using the surface Dice coefficient for evaluation is not a good choice here due to the spherical annotations. We have added a brief explanation for the choice of metrics to the result section

and have updated the method section “Segmentation metrics” to better motivate the choice of metrics and to provide more information to contextualize and interpret the results.

> On page 20, it is stated that the final training data for vesicle segmentation contained 117K annotations, of which 80K derived from manual annotations, and 37K are based on automatically segmented vesicles. More than 30% of the labels used both for training and for testing were generated by SynapseNet. This could bias the process and should ideally be avoided.

The reason for this approach is that vesicles are not densely labeled in our initial manual annotations, because the annotations focused on vesicles close to active zones. This means that, without the additional annotations generated based on automatic segmentation results, we would have non-annotated vesicles in the training volumes, which leads to a worse performance of the trained network. Due to this fact we have adopted a multi stage procedure to add annotations:

- Crop all tomograms with annotations to the minimal bounding box enclosing the labeled vesicles.
- Train an initial version of the vesicle network on a subset of the data that contains crops with almost complete labels (judged by visual inspection).
- Predict with this network on the remaining crops, intersect predictions with ground-truth, visually inspect predictions, add the ones with a good quality to the training pool.
- Repeat this process until all crops show good additional annotations, judged by visual inspection.

This procedure cannot be avoided, as training only on the initial annotations leads to much worse results (see next answer). Please note that such an approach (i.e. training of an initial model, and iteratively refining or extending annotations based on it) is not uncommon to achieve a large segmented dataset. For example, the very large dataset published as part of Segment Anything is created in a similar iterative manner.

> The authors should evaluate the performance of synaptic vesicle segmentation trained solely on the 80K manual annotations. Additionally, they should assess the performance of the model trained with the 117K annotations,

We have performed this experiment. The performance of the model trained only on the 80K manual annotations is significantly worse, due to the fact that its training data contains many vesicles that are not annotated. Below are the results comparing the SynapseNet model (trained on all annotations, same results as in Figure 2), with the model trained only on the manual annotations. Please note that the evaluation results reported here were computed based on the tomograms in the test splits with **all annotations**. Otherwise it is not possible to compute the F1-score or the SBD-score; see the next answer for details.

**Dataset**	**SynapseNet Model (trained on all annotations)**	**Ablation Model (trained only on manual annotations)**

	F1-Score	SBD-Score	F1-Score	SBD-Score
Chemical Fixation	77.8%	63.4%	5.3%	17.2%
Single-Axis TEM Tomo	85.0 +- 3.1%	68.9 +- 4.3%	81.2 +- 5.3%	65.4 +- 5.6%
Dual-Axis TEM Tomo	90.0 +- 1.6%	75.3 +- 3.0%	56.3 +- 14.5%	41.5 +- 13.6%
STEM Tomo	87.6 +- 6.9%	73.6 +- 8.3%	60.0 +- 23.1%	44.4 +- 21.9%
OVERALL	87.0 +- 6.1%	72.5 +- 7.3%	61.3 +- 24.3%	46.7 +- 21.8%

> but the evaluation should be restricted to the subset of vesicles that were manually annotated.

This is not possible. As explained before, the manual annotations do not cover all vesicles. So we can only compute the Recall, but not Precision, F1-Score or SBD, if we limit the evaluation to manually annotated vesicles. Below are the results of this experiment, i.e., evaluation of the SynapseNet and Ablation Model evaluated via the recall of manually annotated vesicles. The recall was computed at an overlap threshold of 50% and the same test splits as before were used. As can be seen, the SynapseNet model achieves a higher recall than the Ablation Model in almost all cases, especially in the overall evaluation.

**Dataset**	**SynapseNet Model (All Annotations)**	**Ablation Model (only manual annotations)**
	**Recall**	**Recall**
Chemical Fixation	88.7%	84.7%
Single-Axis TEM Tomo	98.1 +- 2.0%	95.5 +- 10.2%
Dual-Axis TEM Tomo	97.7 +- 2.5%	90.3 +- 6.45%
STEM Tomo	82.2 +- 22.1%	85.9 +- 18.1%
OVERALL	96.1 +- 7.0%	91.9 +- 10.2%

The motivation for our procedure in the data generation was already clearly stated in the section on “Data Annotation”. We have now added a short summary of the ablation experiments described here to it.

> The authors compare synaptic vesicle segmentation performance of SynapseNet and CryoVesNet. It is not clear how they used CryoVesNet. Did they use only the U-Net or did they also use the postprocessing steps of CryoVesNet? This should be clarified.

We also used the post-processing steps of CryoVesNet. We have now clarified this in the method section “Other Tools for Synaptic Reconstruction”, where we now discuss the comparison to CryoVesNet in more detail to also address the remaining points raised regarding this comparison.

> *CryoVesNet has been trained on a cryo-ET dataset. It is therefore not surprising that it performs much worse on resin-embedded samples than SynapseNet, as the authors write it. It should also be mentioned that the size of CryoVesNet training dataset (1369 vesicles) is about 100 fold smaller than SynapseNet training dataset and that CryoVesNet was trained with fewer epochs.*

Our motivation for comparing to CryoVesNet was not to provide an unbiased comparison between models, but rather to evaluate SynapseNet in the context of other methods for synaptic vesicle segmentation. Out of the methods we are aware of, CryoVesNet was the only one that could be applied to our data in a reliable fashion and that provided ‘reasonable’ results, see also next answers regarding 3D-ART-VeSElecT and the work of Gomez-de-Mariscal et al. We believe that this provides meaningful context to a reader looking to apply methods for synaptic vesicle segmentation to electron tomography, as it indicates that SynapseNet is a superior model for room-temperature electron tomography, whereas CryoVesNet performs better “out-of-the-box” for cryogenic electron tomography, with a similar performance after domain adaptation of SynapseNet.

We acknowledge that we did not explain the systematic disadvantage of CryoVesNet in enough detail before, and have now added a short explanation on this in the result section and an extensive explanation, including a comparison of the training data sets, in the method section “Other Tools for Synaptic Reconstruction”. We have also marked CryoVesNet with a * in Figures 2 and 3 and added a short disclaimer referencing the * and the caveats about the comparison to their captions.

> *In order to ensure a fair comparison, I propose two options: either omit the F1-Scores of CryoVesNet for resin-embedded samples from Figure 2B and Figure 3A for EH and IER, or retrain CryoVesNet using the same training dataset as SynapseNet and include a third column in Figure 2B to present the F1-Scores of the retrained model.*

We would again like to point out the reason for this comparison: evaluate the tools that are available to a user that wants to segment vesicles in electron tomography or electron microscopy. Retraining CryoVesNet would be a major burden to such a user, so our current comparison is the most practically relevant one. We acknowledge that this is not unbiased from a method comparison point of view, but comparing the core segmentation methodology is not the main objective of our paper, as indicated in our previous answer. Due to the large amount of effort required to address the other points raised in this review we could not retrain CryoVesNet. Please note that adapting the data preparation procedure and the training procedure of CryoVesNet in a correct manner would have required significant effort, due to several design

choices in the respective notebooks that appear specific to the CryoVesNet training data.

> *Another interesting comparison could be to train SynapseNet with the training set of CryoVesNet (available on EMPIAR, including the training labels) and evaluate its performance on cryo-ET datasets.*

We have performed this experiment, and have trained our vesicle segmentation model from scratch, i.e. with randomly initialized weights, on the training data of CryoVesNet, using seven tomograms for training, one for validation and leaving out one tomogram for testing. The results of this network, applied both to the test tomogram from the CryoVesNet data and the Cryo ET tomograms from our study are shown below:

**Dataset**	**SynapseNet (CryoVesNet data)**	**SynapseNet (Source)**	**SynapseNet (adapted)**	**CryoVesNet**
Spray CTRL 6 (CryoVesNet)	88.0% (F1)<br>68.2% (SBD)	-	-	-
Cryo-33K	81.6% (F1)<br>69.0% (SBD)	85.2% (F1)<br>79.9% (SBD)	91.7% (F1)<br>87.3% (SBD)	85.6% (F1)<br>79.7% (SBD)
Cryo-64K	77.0% (F1)<br>60.0% (SBD)	77.0% (F1)<br>66.3% (SBD)	89.3% (F1)<br>79.1% (SBD)	89.5% (F1)<br>84.7% (SBD)

We have included the corresponding results for the SynapseNet source vesicle model, the model adapted to the CryoET data, and the CryoVesNet model (from Figure 3 A). As we see, this model obtains good results on the CryoVesNet data domain, but it does not generalize better to other Cryo ET data, compared to the original SynapseNet source model. Given this fact, we have not investigated this model further. The fact that the CryoVesNet model, which was trained on the same data (though potentially using different train/val/test splits) seems to generalize better in this case is interesting, but we didn't have further time to investigate this fact. We have shortly summarized these results in the "Other Tools for Synaptic Reconstruction" section.

> *It would be interesting to compare the performance of synaptic vesicle segmentation of SynapseNet with 3DART-VeSElecT, which is based on classical image analysis. The authors decided not to do it because it requires some manual parameter tuning, which prevents automation. Nonetheless, the authors could test 3DART-VeSElecT with its default parameter values.*

We tried to apply VeSElecT to our tomograms, but couldn't find a way to apply it in a headless fashion (macro recording in Fiji did not work). In order to apply it to many tomograms, headless application is essential for scripting, otherwise these experiments are not feasible in a reasonable amount of time, as each tomogram would need to be processed individually in Fiji.

We can thus not perform these experiments. We now point out this apparent limitation of VeSElecT in the section “Other Tools for Synaptic Reconstruction”.

> *Furthermore Gomez-de-Mariscal et al. have developed a deep-learning-based segmentation of small extracellular vesicles in TEM images (https://doi.org/10.1038/s41598-019-49431-3). It would be also interesting to compare the performance of this approach with SynapseNet for synaptic vesicle segmentation.*

We did not include this method in our initial experiments, as it is trained on quite different data. Extracellular vesicles have a very different organization, compared to synaptic vesicles: most importantly they do not form the clusters that are typical for synapses. We have now applied the method indicated by the reviewer to 2D TEM data from our study. We found that it did not reliably segment vesicles in this dataset. Please note that we have only applied their pre-trained model. Re-training their model is out of scope for our revision, and is also not a very relevant experiment for our target audience due to the effort involved. We have updated the section on “Other Tools for Synaptic Reconstruction” to briefly report our findings.

A more detailed report on our experiments: We have used the model from BioImage.IO (model name: naked-microbe) in DeepImageJ on our sample image for 2D TEM data. As you can see from the screenshot, the model only identifies very few structures, some dense core vesicles and some non-vesicular structures, in the image.

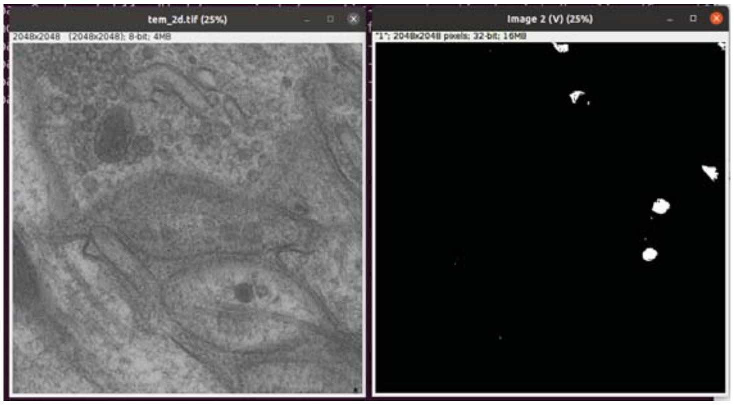

We have verified that the size of the training data of the model roughly matches our sample image, see the screenshot below showing the sample data of the model (left), a region from our data of the same size (middle), and the predictions from the model for its sample data (right). As you can see, the size of the vesicles is roughly similar, so there is no point in resizing the data to improve results.

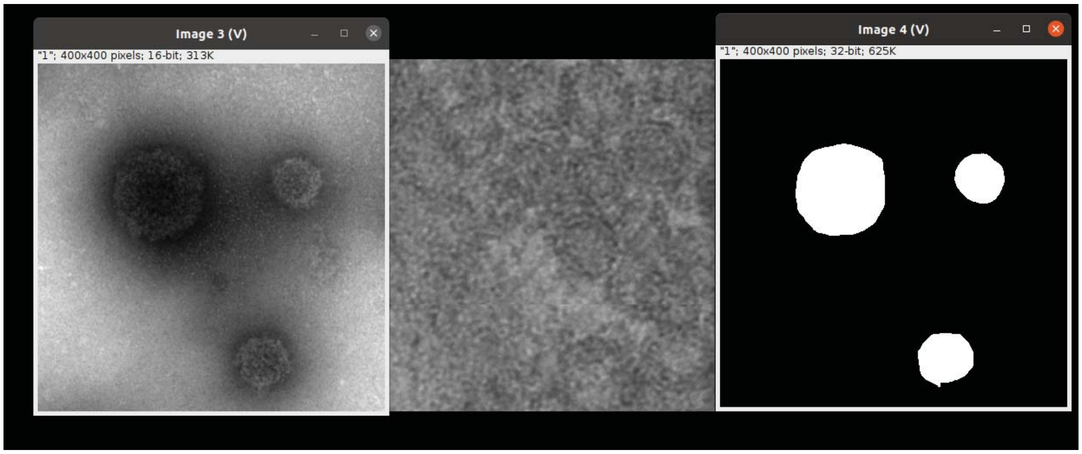

Note that our 2D model works much better for the synaptic 2D TEM data; the screenshot below shows the prediction of the model before domain adaptation.

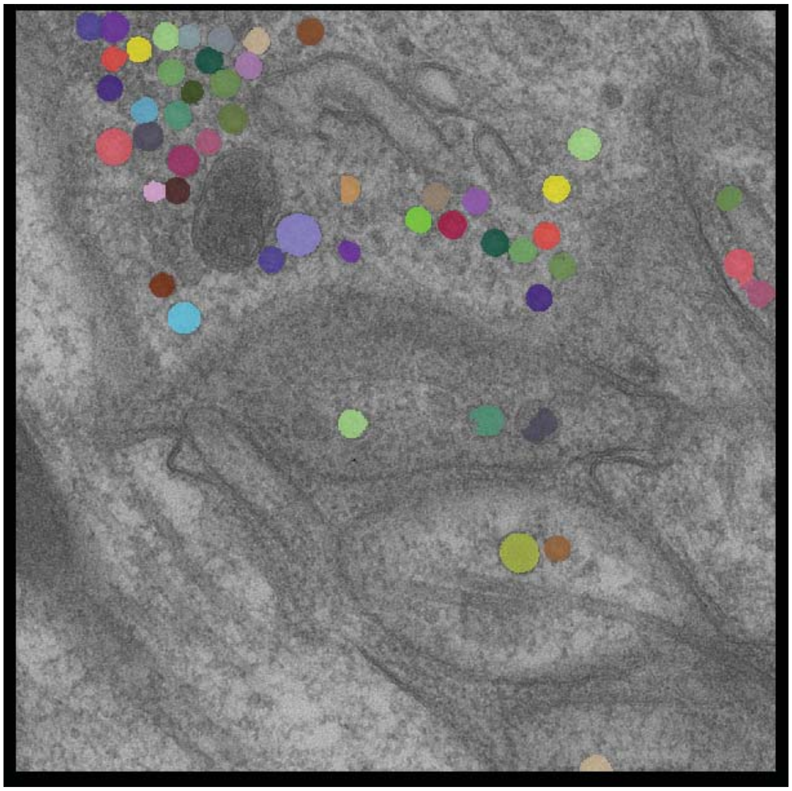

> The segmentation of mitochondria and synaptic compartments were only visually evaluated. This is not sufficient. F1-score and Dice coefficient should be presented.

Both segmentations were initially only qualitatively evaluated due to the limited amount of annotated data. Data annotation poses a major time investment, and we could thus not invest the time to annotate both sufficient mitochondria and compartments in the revision period to provide a thorough investigation in both cases. We have focused on the annotation of mitochondria, for which we now have a separate test set.

In more detail, we have now annotated mitochondria in 5 additional STEM tomograms, which contained a total of 30 mitochondria. This corresponds to a size of about 30% of the training set (29 tomograms, 90 mitochondria). We have used this data as a test set for evaluating our model, where we found it to perform accurately, with a F1-Score of 94.8% and a SBD-Score of 86.9%. We have added these results to the result text and as panel C to Supp. Figure 2.

For the synaptic compartments please note that we have qualitatively evaluated it on a relatively large dataset of 35 STEM tomograms, where we checked 74 terminals. This provides a reasonable evaluation of the quality of this model for STEM data. Furthermore, we have used the model as part of the processing pipeline for the analysis of SNAP/Munc data, showing the utility of the model. Please note that this analysis is now fully automated see next section.

> The benefit of active zone segmentation is not very clear. Dice coefficient is relatively poor (~0.5). On another test set, it is stated that 67% of active zones were found, but in this case, there is no mention of the false positive rate. Could the authors specify an F1-Score? On page 10, it is stated that in 60% of active zones were segmented completely and 40% had to be corrected. Figure 5 compares manually segmented active zones (Fig. 5B and E) with semi-automatically segmented active zones (Fig. 5C and F). They look visually identical but it would be interesting to see the fully automatically segmented AZ for comparison. The authors could indicate how much time/effort is saved by the semi-automatic segmentation of AZ versus fully manual segmentation.

We have significantly improved the active zone model in the revision, by improving the procedure for extracting active zone annotations from IMOD and extending the training data to more tomograms, including tomograms from the endbulb of held (EH) data (from https://www.cell.com/iscience/fulltext/S2589-0042(23)02777-3). We have trained a new version of the active zone model on this data and evaluated it on test sets of four different types of tomograms: chemical fixation, single-axis TEM, STEM, and EH. See Supp. Figure 2B for an overview of these results. We also updated the evaluation metrics, using the surface Dice score, which is better suited to evaluate this kind of segmentation. We have used the surface Dice implementation from https://www.biorxiv.org/content/10.1101/2024.01.05.574336v2.

We found that the active zone segmentation yielded good results for the chemical fixation and TEM tomograms (surface Dice of around 70 %). Both of these types of tomograms contain only a single active zone. For STEM and EH, the scores are lower. This can be explained to a large degree by the fact that these tomograms are much larger, containing multiple active zones. For these tomograms, the active zone model predicts false positives, which lead to a lower surface Dice score. We have shown this by also computing the surface Dice score only for local crops around the true active zones in these tomograms, see the second column in Supp. Figure 2B. Here, the scores improve significantly, especially for STEM tomograms with a score of about 70%. In summary, we find that this model provides accurate segmentations of active zones, while predicting some false positives for large tomograms. In practice, this means that it can be used for full automation in small tomograms, see also next paragraph. For larger tomograms it cannot be used for full automation, but can still significantly speed up analysis by

semi-automation: the user can leverage the predictions to easily find active zones and select only the correctly segmented ones from the model for analysis.

We have also redone the analysis of the SNAP / MUNC tomograms (Section: “Automatic Reconstruction and Analysis”). Here, we found that the new model now enabled fully automatic analysis. This means the correction of active zone segmentations we previously had to perform was no longer necessary. Furthermore, we now find a better agreement of manual and automatic analysis, with both reporting significant differences in the docked phenotype for both SNAP and MUNC knock-outs.

> The discussion states : "This contribution marks the first comprehensive approach for automatic synapse reconstruction from electron micrographs". Although one aspect of the synapse reconstruction is fully automatic (vesicle segmentation), most other aspects are not. Indeed, in addition to active zone segmentation, other structures are semi-automatically segmented: synaptic compartments (mostly split in multiple pieces), presynaptic density in ribbon synapses and to some extent synaptic ribbons. Therefore this sentence, as well as several other sentences, including the title should be modified to reflect the reality of the method.

We already pointed out that the kind of automation possible is dependent on the type of segmentation and analysis, see the third paragraph of the discussion, implying that we did acknowledge this point already. Please also note that the SNAP / MUNC analysis is now automated because we no longer had to correct the active zone segmentations. To acknowledge the comment of the Reviewer, we have added the prefix “(semi-)” to “automated” in the first sentence of the discussion.

> The flow of the manuscript can be difficult sometimes. Many abbreviations used throughout the manuscript (such as IER, EH, ...) are introduced in the Materials and Methods section, which comes at the end of the manuscript, making it difficult to follow the text seamlessly. If the journal permits, it would be helpful to place the materials and methods section before the results section.

The suggested re-structuring does not fit the MBoC guidelines: https://www.molbiolcell.org/info-for-authors#preparing. It is difficult to remove the abbreviations we used, since they do simplify many of the statements and figures. To aid the reader, our manuscript contains explicit reference to the method section to indicate where the data related abbreviations can be found. We have now also added a brief explanation of the most important data abbreviations in the beginning of the result section.

Finally, we would like to point out that the second reviewer found the manuscript well written and understandable.

> Minor comments [...]

We have addressed these points. Below are two specific remarks on the minor comments:

> P. 23. "The networks were for 100,000 iterations and the checkpoint of the epoch with the lowest validation loss was exported." "run" is missing after the networks were. 100,000 epochs is very large. At which iteration was typically the lowest validation loss? If it was early, what was the rational for running so many epochs?

This is a misunderstanding. We trained for 100,000 **iterations**, i.e. batches, not for 100,000 **epochs**.

> P. 23. It would be helpful to illustrate graphically the steps of synaptic vesicle segmentation. Showing an real life example of tomogram region with synaptic vesicles, next to the corresponding prediction maps (boundary and foreground), distance maps, heightmaps, and final results.

We have added this as Supp. Figure 8.

Response to Reviewer #2:

*> * From reading the manuscript, it was not entirely clear whether the Domain Adaptation component is also part of the napari plugin. After reading the documentation, it seems it is not. I suggest that the authors make the distinction between SynapseNet (the method and pre-trained network) and the tool (the napari plugin) more clear. A similar point can be made regarding the fine-tuning (although I do acknowledge that the manuscript mentions that this feature is only available programmatically). Along those lines, I'd be curious to hear whether there are plans to include both features in the plugin, or whether there are practical concerns that prevent it.*

The domain adaptation is not part of the plugin itself, but we offer example scripts and a command line interface (CLI) for it. The reason for this is that long running trainings in the GUI are potentially unstable, so it is a better practice to run this in a script or via CLI. We have clarified this in the manuscript and added better documentation of scripts / CLI to our documentation so that these features are also available to a user without extensive computational expertise. See also the documentation on supervised training and on domain adaptation.

*> * Regarding the Domain Adaptation, I wonder if the available ground-truth annotations could be leveraged to guide the student network, e.g., through an adversarial loss that criticizes solutions that do not look representative of real synapse annotations. Was a strategy like this considered, and if so, are there some preliminary findings that would be worth discussing?*

Such a strategy should be feasible and sounds promising, but would require a significant investment in method development. We cannot address it in the scope of the revision. We have added a short paragraph to the discussion to address advanced ideas for domain adaptation.

> * *Are there any plans to consolidate a large synapse annotation dataset sourced from several contributors in the community? It seems like the authors are in an excellent position to start such an initiative, given the curated datasets they already have.*

We would like to thank the Reviewer for this excellent idea. We have created a section about this in our documentation that outlines the process to submit data and annotations from users. See https://computational-cell-analytics.github.io/synapse-net/synapse_net.html#community-data-submission. We have also updated the discussion to address this point.

RE: Manuscript #E24-11-0519R
TITLE: "SynapseNet: Deep Learning for Automatic Synapse Reconstruction"

Dear Dr. Pape:

I am pleased to accept your manuscript for publication in Molecular Biology of the Cell.

Assaf

Sincerely,
Assaf Zaritsky
Monitoring Editor
Molecular Biology of the Cell

Dear Dr. Pape:

Congratulations on the acceptance of your manuscript! Thank you for publishing your work in Molecular Biology of the Cell (MBoC).

Within 10 days, an unedited PDF of your manuscript will be published on MBoC in Press, an early-release journal version. The date your manuscript appears on this site is the official publication date.

Your copyedited and typeset manuscript will be scheduled for publication in the next available issue of MBoC. Once your paper is ready for review, our production team will notify you. In the summer of 2024, our production provider will introduce an online proofing solution, which will streamline the process of checking and validating author changes. This will result in improved typesetting quality, enhanced digital content, and a faster overall process.

We invite you to submit images related to your accepted manuscript to be considered for the journal cover.
Cover images must contain color, TIF Format
Size image at 7.4" wide x 5.85" deep
RGB and CMYK color (please submit two separate files, one in each format)
300 dpi resolution

Please also send a 1-paragraph description of the image that a general audience could understand. Include with the caption the name and institution of the individual(s) the image should be attributed to. Please provide this description in a Word file. You can send the file via email or by your favorite file transfer service to mboc@ascb.org.

Authors of Articles and Brief Communications are also encouraged to create a short video abstract to accompany their article when it is published. These video abstracts, known as Science Sketches, are up to 2 minutes long and will be published on YouTube and then embedded in the article abstract. Science Sketch Editors on the MBoC Editorial Board will guide you as you prepare your video. Information about how to prepare and submit a video abstract is available at www.molbiolcell.org/science-sketches. Please contact mboc@ascb.org if you are interested in creating a Science Sketch.

We look forward to publishing your paper in MBoC.
Sign up for MBoC's new content alerts here https://tinyurl.com/3bcsyk3e

Sincerely,

Eric Baker
Journal Production Manager
MBoC Editorial Office
mbc@ascb.org
